# Efficacy and Safety of Multiple Dosages of Fostamatinib in Adult Patients With Rheumatoid Arthritis: A Systematic Review and Meta-Analysis

**DOI:** 10.3389/fphar.2019.00897

**Published:** 2019-08-14

**Authors:** Yaqi Kang, Xinrui Jiang, Dalian Qin, Long Wang, Jing Yang, Anguo Wu, Feihong Huang, Yun Ye, Jianming Wu

**Affiliations:** ^1^School of Pharmacy, Southwest Medical University, Luzhou, China; ^2^Institute of Cardiovascular Research, The Key Laboratory of Medical Electrophysiology, Ministry of Education of China, Collaborative Innovation Center for Prevention and Treatment of Cardiovascular Disease of Sichuan Province, Medical Key Laboratory for Drug Discovery and Druggability Evaluation of Sichuan Province, Luzhou Key Laboratory of Activity Screening and Druggability Evaluation for Chinese Materia Medica, Luzhou, China; ^3^Department of Pharmacy, Affiliated Hospital of Southwest Medical University, Luzhou, China

**Keywords:** fostamatinib, rheumatoid arthritis, ACR 20, DAS23-CRP, SF-36, HAQ-DI response, systematic review

## Abstract

**Background:** Rheumatoid arthritis is a type of systemic and complex autoimmune other disease characterized by chronic joint inflammation. Spleen tyrosine kinase (Syk) inhibitors are regarded as an effective alternative to existing drugs for the treatment of this disease. However, studies evaluating fostamatinib, a new Syk inhibitor, are either invalid or insufficient. Through a systematic review and meta-analysis, we evaluated the efficacy and safety of fostamatinib at different dosages in rheumatoid arthritis patients that display an inadequate response to methotrexate or disease-modifying antirheumatic drugs.

**Methods:** Randomized controlled trials published between January 2000 and November 2018 were retrieved from PubMed, Embase, Medline, Web of Science, and The Cochrane Library. We also searched a relevant website (www.clinicaltrials.gov) for retrieval of unpublished data. These studies compared different dosages of fostamatinib to placebo, including the intake of 100 mg fostamatinib twice per day (bid) for 4 weeks followed by 150 mg once per day (qd) vs. the intake of 100 mg bid.

**Results:** Two investigators analyzed 11 randomized placebo-controlled trials consisting of 3,680 patients. Compared to placebo, fostamatinib resulted in an obvious reduction in the American College of Rheumatology 20% response standard [weighted mean difference (WMD) 1.96, 95% confidence interval (CI) [1.46, 2.61], P < 0.001] and disease activity score < 2.6 (WMD 4.70, 95% CI [3.14, 7.03], P < 0.001). Regarding safety, the incidence of serious adverse reactions was higher in the fostamatinib group than in the placebo group [risk ratio (RR) 2.10, 95% CI [1.57, 2.80], P < 0.001]. The same was true for other adverse events [RR 1.63, 95%CI [1.33, 2.01], P < 0.001].

**Conclusions:** Fostamatinib is an effective and safe therapeutic medicine administered to patients with rheumatoid arthritis over 24 weeks. It can alleviate the degree of swelling and inflammation of the joints. Furthermore, 100 mg bid can be considered the most beneficial regimen over a 24-week period. More data are however needed to clarify the incidence of other adverse events and serious adverse reactions.

## Introduction

### Rationale

Rheumatoid arthritis (RA) is one of the world’s most common chronic inflammatory joint diseases (Smolen et al., 2016) caused by the innate and adaptive immune systems. Initially, it is mainly characterized by a chronic, joint synovial inflammation, which is a type of systemic autoimmune dysfunction (Cecchi et al., 2018) that ultimately results in pathological deformities of the joint (Cecchi et al., 2018). Although genetic factors have been estimated to be the main cause (about 50%) of RA, environmental factors, female sex hormones, and infections may also act as a trigger for RA (Scott et al., 2010). The prevalence of RA is relatively stable at 0.5–1.0% of adults in developed countries with 5–50 per 100,000 of incident cases annually (Silman and Pearson, 2002; Scott et al., 2010). Findings of population-based research show that RA is more frequently observed in women and elderly people, with its highest prevalence in women older than 65 years. This suggests that hormonal factors may also play a pathogenic role (Scott et al., 2010). Not only does quality of life decreases and the risk of co-infection increases, but also the working ability of patients with RA reduces (Cross et al., 2014). RA therefore places a heavy burden on society and individuals, warranting the establishment of an early diagnosis and treatment to reduce and prevent subsequent damages.

Existing studies have shown that the pathogenesis of RA is related to the release of interleukin (IL)-1, IL-6, IL-17, IL-23, and tumor necrosis factor-α (TNF-α) (Karmakar et al., 2010). To add, IL-10, transforming growth factor-β (TGF-β), and IL-6 release in osteoclasts promotes the progression of inflammation (Lam et al., 2000; Li et al., 2010). Nevertheless, the exact pathogenesis of RA is yet to be revealed. Through experimental models of arthritis, researchers (Cecchi et al., 2018) revealed that neutrophils are important players in the progression of the disease. Several types of drugs for RA are currently available: 1) traditional disease-modifying antirheumatic drugs (DMARDs) such as sulfasalazine and methotrexate (MTX); 2) biologic DMARDs such as TNF inhibitors, abatacept, and rituximab; and 3) glucocorticoids. However, all three categories of therapies may result in a lack of or an inadequate response. Spleen tyrosine kinase (Syk) inhibitors are therefore considered as an effective alternative to existing drugs.

Syk is a vital non-receptor-type protein tyrosine kinase (PTK) that activates downstream MAPKs and the PI3K pathway to increase the production of IL-6 and matrix metalloproteinase (MMP). Syk is still present in patients with RA synovitis and its activated form plays an important role in the production of fibroblast-like synoviocytes induced by TNF-α (Pine et al., 2007). Fostamatinib (R935788) is the prodrug of R406 that acts as a potent Syk inhibitor (Scott, 2011). Fostamatinib has excellent physiochemical properties and can be rapidly and extensively metabolized to R406 by intestinal alkaline phosphatase, allowing easy absorption of the highly hydrophobic R406 (Scott, 2011). Fostamatinib was demonstrated to have potent anti-inflammatory effects through selectively abrogating the B-cell receptor signaling pathway, suppressing joint swelling, joint synovitis, bone erosion, and pannus formation (Liu and Mamorska-Dyga, 2017). This finding has also been confirmed in rats (Pine et al., 2007). Experiments in healthy volunteers have also demonstrated that fostamatinib is appropriate for clinical development (Baluom et al., 2013).

### Objectives

To evaluate the efficacy and safety of multiple doses of fostamatinib in patients with active RA through a systematic review and meta-analysis.

### Research Question

To date, many clinical trials on fostamatinib have been completed and some have evaluated its safety and effectiveness (Kunwar et al., 2016). However, a study comparing the efficacy of multiple dosages and different administration methods has not been performed. This is due to limitations such as outcome indicators and the inability to evaluate the quality of life of patients. Nonetheless, a study identified a significant improvement using the health assessment questionnaire (HAQ) and physical component scores (PCS) in the group administered fostamatinib (Kawalec et al., 2013). We conducted a systematic review and meta-analysis to evaluate the efficacy and safety of multiple doses of fostamatinib in patients with active RA.

## Methods

### Study Design

A meta-analysis based on articles and randomized controlled trials (RCTs) related to fostamatinib for the treatment of RA selected from various databases.

### Systematic Review Protocol

Two investigators (K.Y.Q and J.X.R) independently reviewed the title and abstract of studies related to fostamatinib for the treatment of RA and selected RCTs. Published or unpublished RCTs were searched in databases without language restriction. All selected studies were read in detail and those that met the inclusion criteria were selected for final analysis. All trials had the following conditions (**Table 1**):

1. Patients: any race, older than 18 years, and diagnosed with inadequate response to MTX or DMARDs for RA.
2. Interventions: use of fostamatinib in multiple dosages as therapy, with an intervention duration of at least 6 weeks.
3. Comparison: A) multiple doses of fostamatinib compared to placebo at 24 weeks. B) 100 mg twice per day (bid) for 4 weeks followed by 150 mg once per day (qd) compared to 100 mg bid.
4. Outcomes: the following indicators were reported from the studies: a) American College of Rheumatology response criteria of 20, 50, 70 percentage (ACR20/50/70); b) American College of Rheumatology index of RA improvement (ACRn); c) Disease activity score based on a count of swollen and tender joints (out of 28 joints), C-reactive protein (blood test measures of inflammation) and the patient’s own assessment (DAS28-CRP). DAS28-CRP < 2.6 or DAS28-CRP ≤ 3.2, DAS28-CRP by using European League Against Rheumatism (EULAR) response; d) SF-36, which is a 36-item short form health survey, evaluation of the indicators of a healthy quality of life. PCS: Physical component scores, a scale of 0 to 100. MCS: Mental component scores, a scale of 0 to 100. A higher score can represent a better quality of life; e) HAQ-DI ≥ 0.22: HAQ - disability index response which compares change (≥0.22) from baseline; and f) serious adverse events (SAEs) and other AEs. Exclusion criteria included non-randomized trials, animals, healthy volunteers, case reports, or conference abstract.

**Table 1:** PICOS criteria for inclusion and exclusion of studies.

Parameter	Inclusion criteria	Exclusion criteria
*Patients*	Adults of any race, older than 18 years; Diagnosed with rheumatoid arthritis and inadequate response to treatment with MTX or DMARDs.	Patients under the age of 18; Females who are pregnant or breast feeding; Healthy people without rheumatoid arthritis.
*Intervention*	Fostamatinib in 50 mg bid, 75 mg bid, 100 mg bid dosages as therapy; Duration of at least 6 weeks.	Other doses of fostamatinib, such as 150 mg qd, 100 mg qd.
*Comparator*	Multiple doses of fostamatinib vs. placebo. Fostamatinib 100 mg twice per day (bid) for 4 weeks followed by 150 mg once per day (qd) vs. fostamatinib 100 mg bid.	Comparing with adalimunab.
*Outcomes*	Primary outcome: ACR 20; Second outcomes: ACR50/70 and ACRn, DAS28-CRP<2.6 or DAS28-CRP ≤ 3.2, DAS28-CRP EULAR ResponseSF-36 in PCS and SF-36 in MCS, HAQ-DI ≥ 0.22; Adverse effects or complications related to the use of fostamatinib.	Studies without defined clinical outcomes; Research only based on Radiography.
*Study design*	Randomized controlled trials.	Review, case reports, no-human studies, conference abstract, pharmacokinetic studies.

PICOS, patients, intervention, comparator, outcomes, study design; RCTs, Randomized clinical trials; qd, once per day. ACR20/50/70, American College of Rheumatology response criteria of 20, 50, 70 percentage; ACRn, American College of Rheumatology index of RA improvement; DAS28-CRP, Disease activity score based on a count of swollen and tender joints (out of 28 joints), C-reactive protein (blood test measures of inflammation) and the patient’s own assessment; SF-36, 36-item short form health survey, evaluation of the indicators of a healthy quality of life; PCS, Physical component scores, a scale of 0 to 100; MCS, Mental component scores, a scale of 0 to 100; HAQ-DI ≥ 0.22, HAQ—disability index response that compares change (≥0.22) from baseline.

### Search Strategy

**Figure 1:**
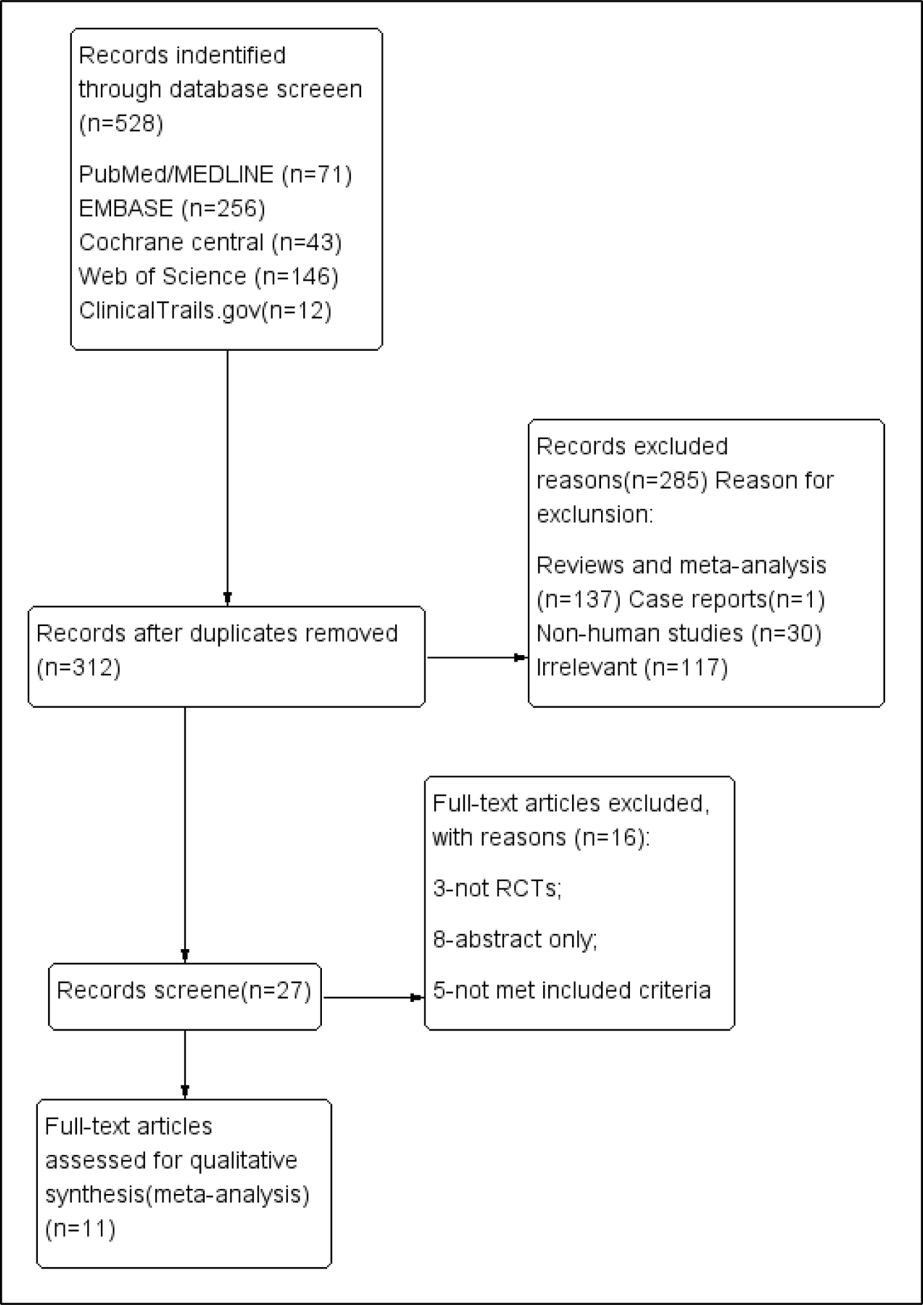
PRISMA flow diagram of the screening and selection process used in the study.

We systematically searched PubMed, Medline, Embase, Web of Science, the Cochrane Library, and Clinical Trials (http://www.clinicaltrials.gov), without language restriction, from their inception to November 2018, (**Figure 1**). We used Mesh database and the following search queries: (((((R788) OR fostamatinib) OR “fostamatinib” [Supplementary Concept])) AND (((“Arthritis, Rheumatoid”[Mesh]) OR rheumatoid arthritis) OR RA). The PROSPERO registration number is “CRD42018117737.”

## Data Extraction

Two investigators (YK and XJ) extracted the summary characteristics of the included studies (study design, number of patients, trial interventions, and outcomes), and participants’ baseline characteristics (age, sex, race, background therapy, and locations). Sponsors were also considered.

### Data Analysis

All primary and second outcomes were analyzed using RevMan 5.3 software (Nordic Cochrane Center, Copenhagen, Denmark; http://www.cochrane.org/). Odds ratio (OR) was calculated with 95% confidence intervals (CIs) for dichotomous data. For adverse events, risk ratio (RR) was computed with 95% CIs. Furthermore, we used weighted mean difference (WMD) with 95% CIs for continuous data. I^2^ was calculated through statistics to estimate heterogeneity. If I^2^ was < 50%, a fixed-effect model with the analyses conducted by the Mantel-Haenszel method was accepted by the two investigators; otherwise, the random-effect model was adopted. Sensitivity analysis was also adopted to examine the possible influence of some of the single studies excluding possible extreme observations. Risk of bias was derived using Cochrane Collaboration’s tool (Higgins et al., 2011) and was estimated separately for different outcomes of interest when considered appropriate by the investigators. Based on the limitations of the research design, the directness, consistency, accuracy, and publication bias of the evidence, the overall confidence of each result was assessed by the quality of evidence as determined by the Grades of Recommendation, Assessment, Development and Evaluation (GRADE) assessment (Atkins et al., 2004). GRADE Pro Software (2014; www.gradepro.org) was used to separately score and chart these indices. All decisions to reduce or improve the quality of evidence were reasonable and presented in the evidence profile. Evidence summary table was in accordance with the GRADE guidelines.

## Results

### Study Selection and Characteristics

We identified 528 publications in five databases at https://www.clinicaltrials.gov/. After excluding duplicate entries, 312 publications were retained. After the exclusion of 137 reviews or meta-analyses, 30 non-human studies, irrelevant data, and 117 irrelevant articles, 27 were retained for further assessment. Only one case report was found. Finally, 11 RCTs (n = 3,680) met the final inclusion criteria for our meta-analysis (https://www.clinicaltrials.gov/1). Among them, the study by Waterton (4SS) was identified to be a sub-study of that of Taylor et al. (2015). To add, two of the clinical trials [(Weinblatt et al., 2010) and (Weinblatt et al., 2013)] shared the same NCT number (NCT 00665925).

Summary characteristics of the included studies are shown in **Table 2**. Patient characteristics in the included studies are provided in **Supplementary Table 1**. All studies were complete and the relevant data were published. There were nine published studies. Information for two clinical trials was found online. All included trials were randomized, double blind, placebo-controlled trials. Three trials lasted 1 to 12 weeks while eight trials lasted 1 to 24 weeks.

**Table 2:** Summary characteristics of included studies.

Reference	Study design	Patients (N)	Trial interventions	Outcomes
Weinblatt et al. (2014)	1:1:1 Randomized double-blind Study start: September 2010	918	Intervention: Fostamatinib: oral treatment dose of 100 mg twice daily or 100 mg twice daily/150 mg once daily for 52-week treatment period Control: Placebo: oral treatment of 24 weeks twice daily then followed by fostamatinib 100 mg twice daily for 52-week treatment period	Primary: ACR20 at 24 week; Secondary: ACR50/ACR70/ACRn at 24week; DAS28-CRP < 2.6 and EULAR response at 12/24 Week; SF-36 - comparison of the change in PCS/MCS and HAQ-DI response at 24 week; Safety: Adverse events
*NCT 01197534*	1:1:1 Randomized double-blind Study start: September 2010	908	Intervention: Fostamatinib: oral treatment dose of 100 mg twice daily or 100 mg twice daily/150 mg once daily for 52-week treatment period Control: Placebo: oral treatment of 24 weeks twice daily then followed by fostamatinib 100 mg twice daily for 52-week treatment period	Primary: ACR20 at 24 week Secondary: ACR50/ACR70/ACRn at 24week; DAS28-CRP< 2.6 and EULAR response at 12/24 week; SF-36 comparison of the change in PCS/MCS and HAQ-DI response at 24 week; Safety: Adverse events
Genovese et al. (2014)	1:1:1 Randomized double-blind Study start: September 2010	322	Intervention: Fostamatinib: oral treatment dose of 100 mg twice daily or 100 mg twice daily/150 mg once daily for 24-week treatment period Control: Placebo: oral treatment of 24-week treatment period twice daily treatment period	Primary: ACR20 at 24 week Secondary: ACR50/ACR70/ACRn at 24week; DAS28-CRP < 2.6/< = 3.2/EULAR response at 12/24 week; SF-36 comparison of the change in PCS/MCS and HAQ-DI response at 24 weeks Safety: Adverse events
Taylor et al. (2015)	1:1:2 Randomized double-blind Study start: December 2010	154	Intervention: Fostamatinib: oral treatment dose of 100 mg twice daily, 100 mg twice daily/150 mg once daily for 24-week treatment period Control: Placebo: oral treatment of 6 weeks twice daily then fosta 100 mg twice daily or Placebo 6 weeks then fosta 100 mg twice daily/150 mg once daily for 24-week treatment period	Primary: DAS28-CRP Score at week 6 Secondary: DAS28-CRP EULAR response at 6 weeks; ACR20/50/70/ACRn at 6–24 weeks; SF-36 comparison of the change in PCS/MCS at 24 weeks; HAQ-DI response at 6/24 weeks Safety: Adverse events
Waterton et al. (2017)	1:1 Randomized double-blind Study start: March 2014	62	Intervention: Fostamatinib: oral treatment dose of 100 mg twice daily, 100 mg twice daily/150 mg once daily for 24-week treatment period Control: Placebo: oral treatment of 6 weeks twice daily then fosta 100 mg twice daily or Placebo 6 weeks then fosta 100 mg twice daily/150 mg once daily for 24-week treatment period	DAS-CRP score at 6 weeks Safety: Adverse events
*NCT 01569074*	1:1:1:1:1 Randomized double-blind Study start: April 2012	163	Intervention: Fostamatinib: oral treatment dose of 100 mg/75 mg/50 mg twice daily or 100 mg twice daily/150 mg once daily for 12-week treatment period Control: Placebo: oral treatment of 12-week twice daily treatment period	Primary: ACR20 at 12 weeks Secondary: ACR50/70/ACRn at 12 weeks; DAS28-CRP< = 3.2 and EULAR response at 12 weeks; SF-36 comparison of the change in PCS/MCS and HAQ-DI response at 12/24 weeks; Safety: Adverse events
Kitas et al. (2014)	1:1 Randomized double-blind Study start: March 2012	135	Intervention: Fostamatinib: oral treatment dose of 100 mg twice daily for 4-week treatment period Control: Placebo: oral treatment of 4-week twice daily treatment period	DAS28-CRP improvement at 4 weeks Safety: Adverse events
Weinblatt et al. (2008)	1:1:1 Randomized double-blind Study start: May 2006	189	Intervention: Fostamatinib: oral treatment dose of 50 mg/100 mg twice daily for 12-week treatment period Control: Placebo: oral treatment of 12-week twice daily treatment period	Primary: ACR20 response rate at 3 months Secondary: ACR 50/70 at 12 weeks; DAS-CRP score at 12 weeks; Safety: Adverse events
Weinblatt et al. (2010)	1:1 Randomized double-blind Study start: May 2008	305	Intervention: Fostamatinib: oral treatment dose of 100 mg twice daily for 6-month treatment period Control: Placebo: Placebo: oral treatment of either once daily, or twice daily for 6-month	Primary: ACR20 at 6 months Secondary: ACR 50/70/ACRn at 3/6 months; DAS28-CRP < 2.6/< = 3.2 at 12/24 weeks; Safety: Adverse events
Weinblatt et al. (2013)	1:1 Randomized double-blind Study start: May 2008	305	Intervention: Fostamatinib: oral treatment dose of 100 mg twice daily for 6-month treatment period Control: Placebo: Placebo: oral treatment of either once daily, or twice daily for 6-month	SF-36 comparison of the change in PCS/MCS at 24 weeks; Safety: Adverse events
Genovese et al. (2011)	2:1 Randomized double-blind Study start: April 2008	219	Intervention: Fostamatinib: oral treatment dose of 100 mg twice daily for 3-month treatment period Control: Placebo: oral treatment of twice daily for 3-month treatment period	Primary: ACR20 response at 3 months; Secondary: ACR 50/70/ACRn at 3 months; DAS28-CRP < 2.6/< 3.2 at 3 months Safety: Adverse events

DB: double-blind.

Each clinical trial was based on an inadequate or ineffective response to MTX (Weinblatt et al., 2014) or DMARDs. Hence, investigators compared 100 mg of the drug to placebo. To derive the optimal dosage regimen in the present meta-analysis, different dosing regimens were selected, six of which were 100 mg bid for 4 weeks followed by 150 mg qd. In addition to a 100 mg bid only, we also selected dosages below 100 mg bid (50 and 75 mg bid).

### Risk of Bias

**Figure 2:**
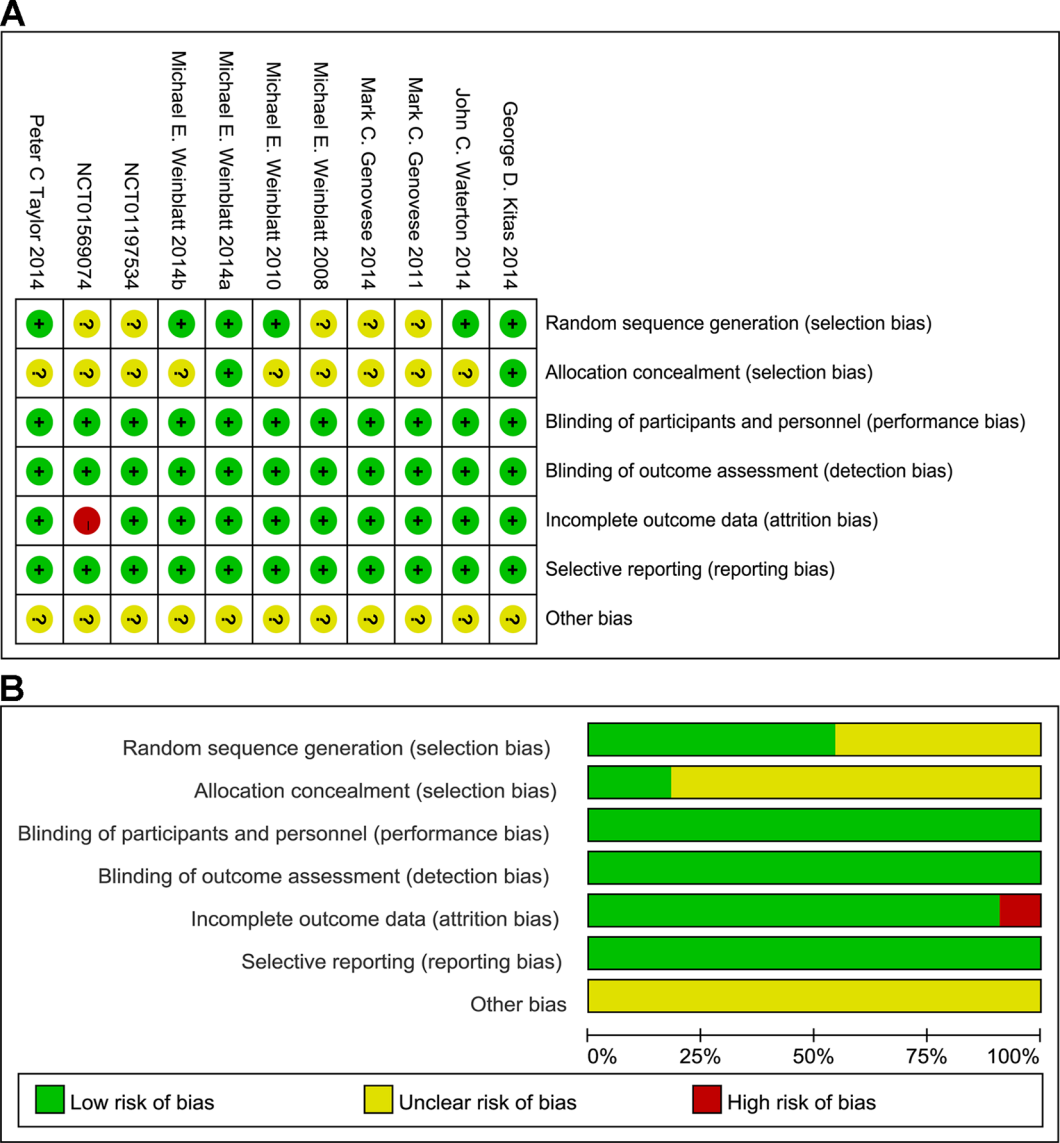
Summary **(A)** and graph **(B)** of the risk of bias in the included trials by Cochrane risk of bias tool. Assessments were based on the reviewers’ judgment of each domain.

Seven of the 11 published trials were judged to be of high quality, but were sponsored by AstraZeneca and Rigel Pharmaceuticals. All included studies were randomized, double-blind, placebo-controlled studies with low risk of bias as evaluated by The Cochrane Collaboration’s tool for assessing risk of bias (**Figure 2**). **Supplementary Table 2** summarizes the confidence findings for the GRADE estimates.

### ACR

#### ACR20/50/70 Response

**Figure 3:**
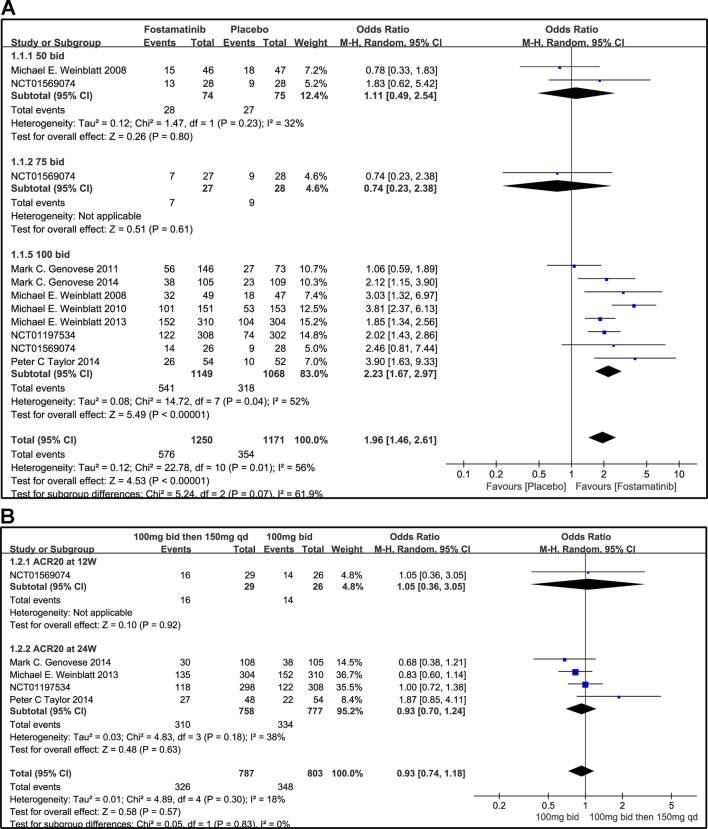
Forest plots for the effect of multiple doses on ACR20 at different time points. **(A)** Subgroups administered multiple doses (50, 75, and 100 mg bid) of fostamatinib vs. placebo at 24 weeks from baseline; **(B)** 100 mg bid for 4 weeks followed by 150 mg bid vs. 100 mg bid at 12 and 24 weeks.

**Figure 4:**
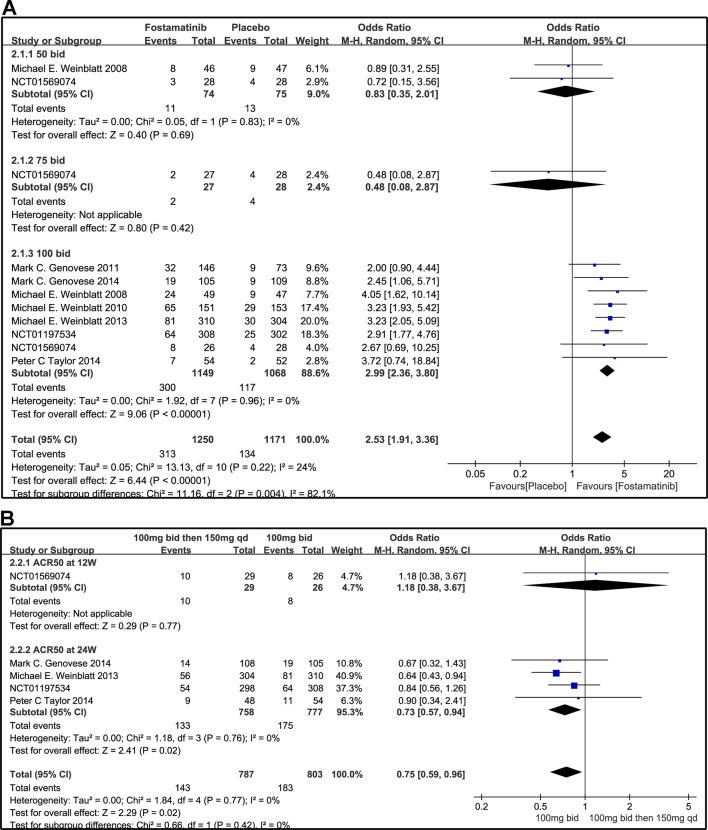
Forest plots for the effect of multiple doses on ACR50 at different time points. **(A)** Subgroups administered multiple doses (50, 75, and 100 mg bid) of fostamatinib vs. placebo at 24 weeks from baseline; **(B)** 100 mg bid for 4 weeks followed by 150 mg bid vs. 100 mg bid at 12 and 24 weeks.

**Figure 5:**
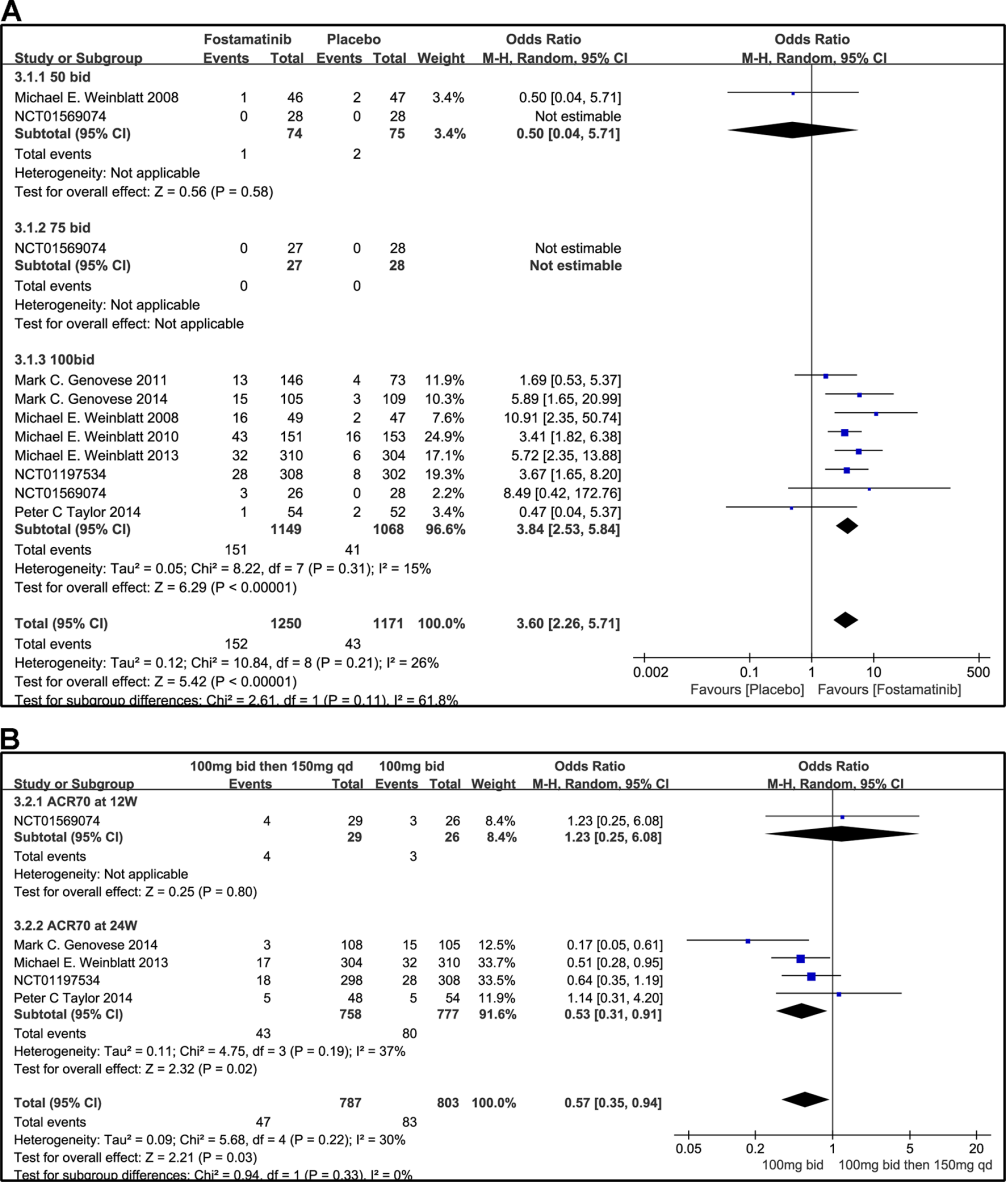
Forest plots for the effect of multiple doses on ACR70 at different time points. **(A)** Subgroups administered multiple doses of fostamatinib vs. placebo at 24 weeks from baseline; **(B)** 100 mg bid for 4 weeks followed by 150 mg bid vs. 100 mg bid at 12 and 24 weeks.

Compared to placebo, fostamatinib achieved a more effective ACR 20/50/70 response (WMD 1.96, 95% CI [1.46, 2.61], P < 0.00001; WMD 2.53, 95% CI [1.91, 3.36], P < 0.00001; WMD 3.60, 95% CI [2.26, 5.71], P < 0.00001, respectively; **Figures 3A**, **4A**, and **5A**). To add, the response efficiency of fostamatinib 100 mg bid was significantly higher than that of the placebo for ACR20/50/70 response (WMD 2.23, 95% CI [1.67, 2.97], P < 0.00001; WMD 2.99, 95% CI [2.36, 3.79], P < 0.00001; WMD 3.84, 95% CI [2.53, 5.84], P < 0.00001, respectively). However, an important outcome indicator, ACR20, had high heterogeneity despite subgroup analysis (I^2^ = 52% for fostamatinib 100 mg bid). With the removal of the study by Weinblatt et al. (2010) due to its better size effect compared to other trials, heterogeneity and effect size of ACR20 for fostamatinib 100 mg bid were significantly reduced (WMD 1.98, 95% CI [1.55, 2.53], P < 0.00001; I^2^ = 26%). An explicit difference between fostamatinib 50 mg (WMD 1.11, 95% CI [0.49, 2.54], P = 0.80; WMD 0.83, 95% CI [0.35, 2.01], P = 0.69; WMD 0.50, 95% CI [0.04, 5.71], P = 0.58, respectively) and 75 mg bid and placebo (WMD 0.74, 95% CI [0.23, 2.38], P = 0.42) (**Figures 3A**, **4A**, and **5A**) was not found.

**Figure 3B** indicates that for ACR20 response (WMD 0.93, 95% CI [0.74, 1.18], P = 0.57), a significant difference was not found between fostamatinib 100 mg followed by 150 mg qd and fostamatinib 100 mg bid at both 12 and 24 weeks. However, the statistical results for ACR50 (**Figure 4B**) and ACR70 (**Figure 5B**) response indicated that fostamatinib 100 mg bid had a better efficacy than fostamatinib 100 mg followed by 150 mg qd (WMD 0.75, 95% CI [0.59, 0.96], P = 0.02 and WMD 0.57, 95% CI [0.35, 0.94], P = 0.03).

#### ACRn Score

**Figure 6:**
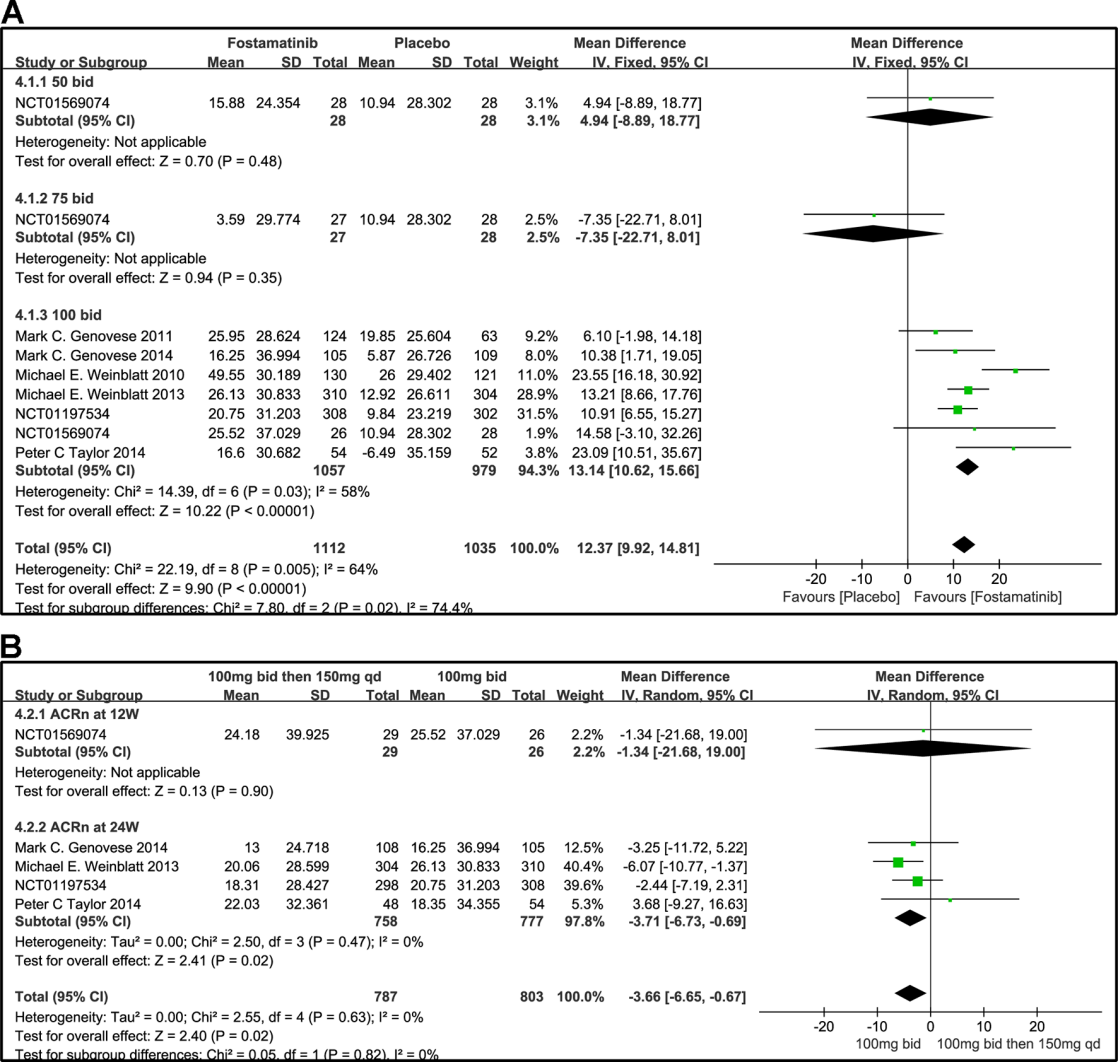
Forest plots for the effect of multiple doses on ACRn at different time points. **(A)** Subgroups administered multiple doses (50, 75, and 100 mg bid) of fostamatinib vs. placebo at 24 weeks from baseline; **(B)** 100 mg bid for 4 weeks followed by 150 mg bid vs. 100 mg bid at 12 and 24 weeks.

Based on the statistical results, despite a relatively high heterogeneity (I^2^ = 64%), ACRn score of fostamatinib was significantly higher than that of placebo (WMD 12.37, 95% CI [9.92, 14.81], P < 0.00001; **Figure 6A**). To add, subgroup analysis did not reduce this high level of heterogeneity at different doses. Subgroup analysis, however, revealed that there was no significant difference between fostamatinib 50 mg (WMD 4.94, 95% CI [-8.89, 18.77], P = 0.48) or 75 mg bid (WMD -7.35, 95% CI [-22.71, 8.01], P = 0.35) and placebo. In contrast, a statistically significant increase in the ACRn score was observed in the fostamatinib 100 mg bid group compared to placebo (WMD 13.14, 95% CI [10.62, 15.66], P < 0.00001), and this was accompanied by high heterogeneity (I^2^ = 58%). The ACRn score of fostamatinib 100 mg followed by 150 mg qd was lower than that of fostamatinib 100 mg bid (WMD -3.66, 95% CI [-6.65, -0.67], P = 0.02) (**Figure 6B**).

### DAS28-CRP

#### DAS28-CRP < 2.6 and DAS28-CRP ≤ 3.2

**Figure 7:**
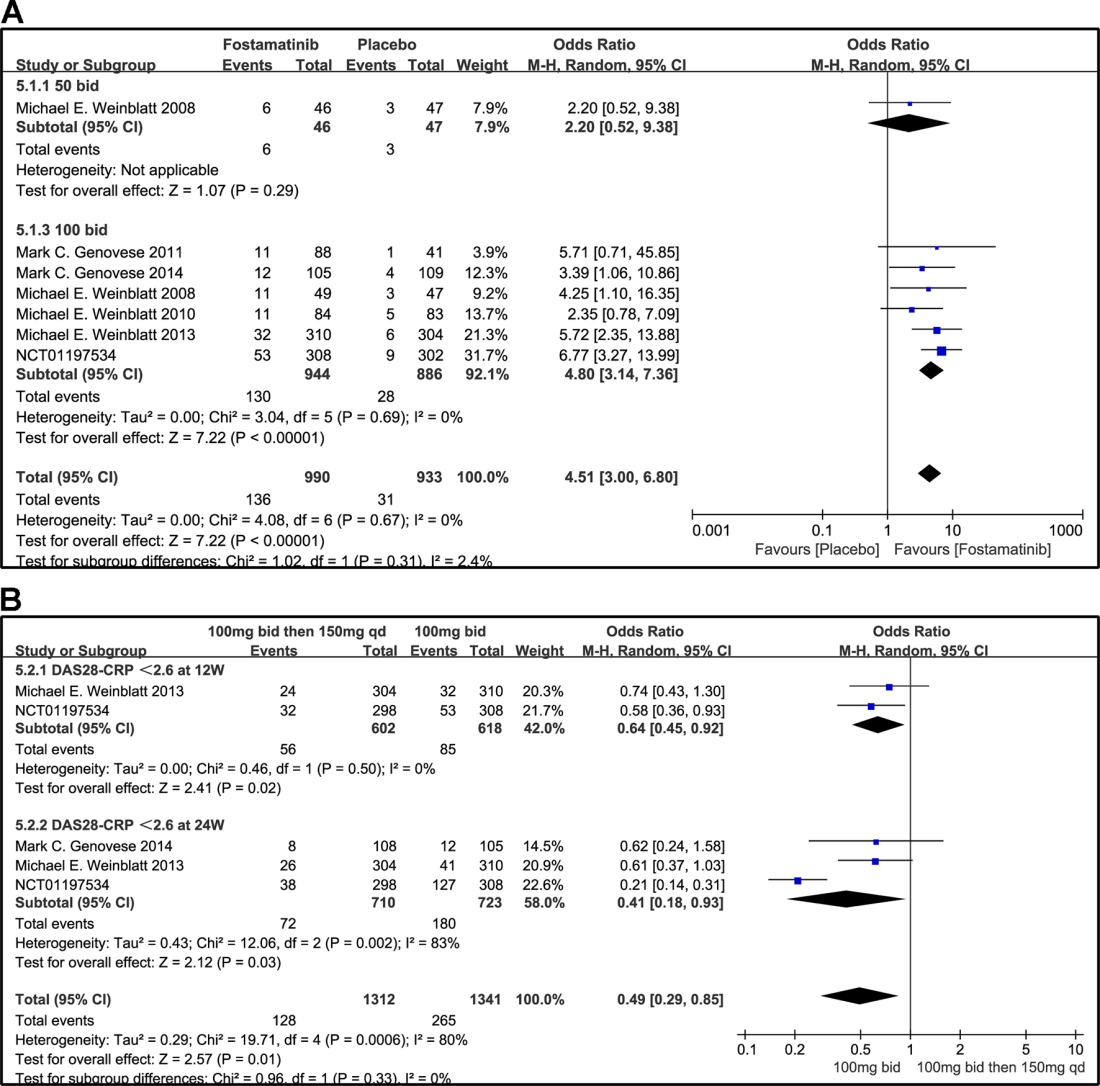
Forest plots for the effect of multiple doses on DAS28-CRP < 2.6 at different time points. **(A)** Subgroups administered multiple doses (50 and 100 mg bid) of fostamatinib compared to placebo at 24 weeks from baseline; **(B)** 100 mg bid for 4 weeks followed by 150 mg bid vs. 100 mg bid at 12 and 24 weeks.

**Figure 8:**
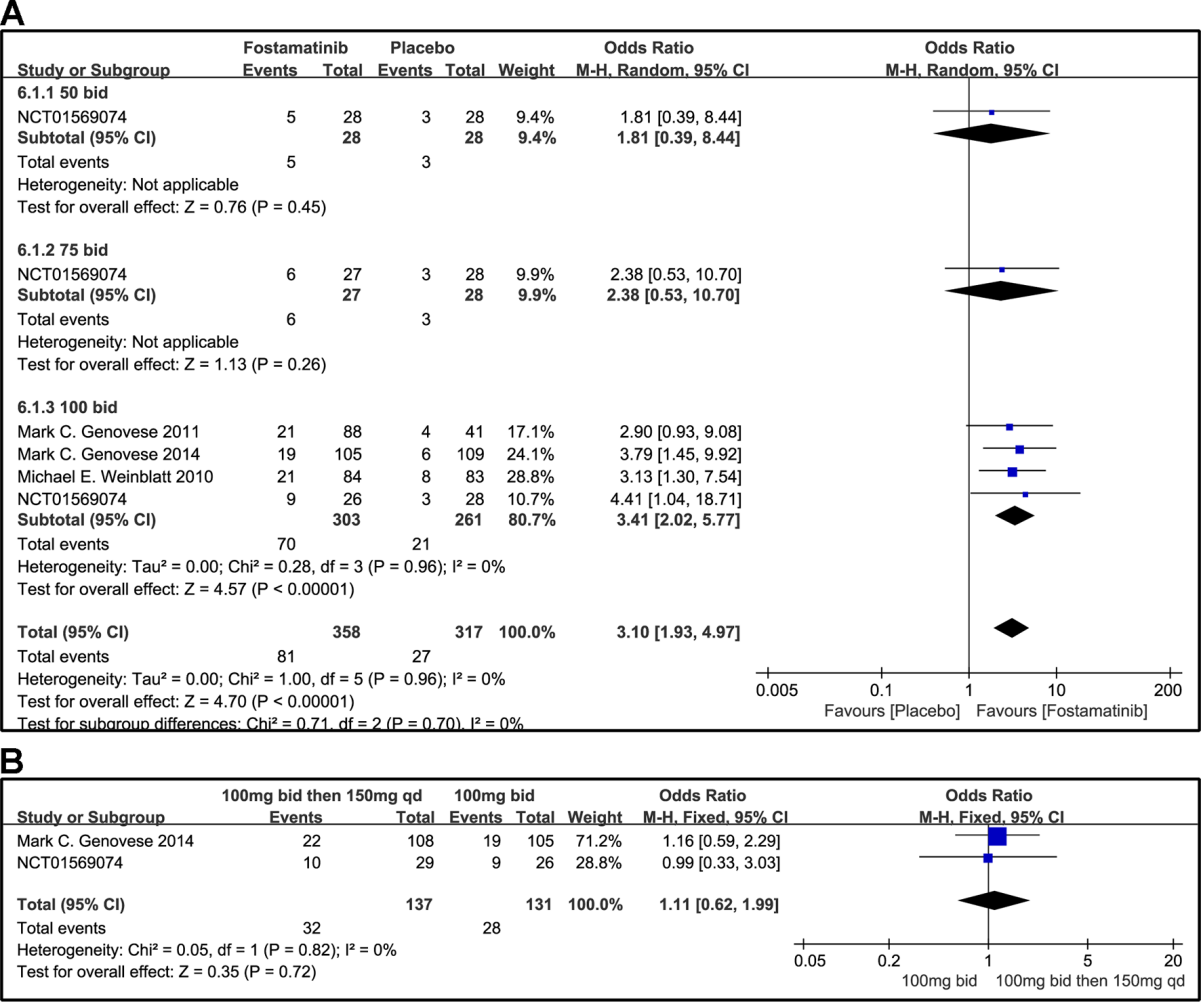
Forest plots for the effect of multiple doses on DAS28-CRP ≤ 3.2 at different time points. **(A)** Subgroups administered multiple doses (50, 75, and 100 mg bid) of fostamatinib compared to placebo at 24 weeks; **(B)** 100 mg bid for 4 weeks followed by 150 mg bid vs. 100 mg bid at 12 and 24 weeks.

A DAS28-CRP score <2.6 indicated remission of RA symptoms while ≤ 3.2 indicated low disease activity. Statistical results showed that fostamatinib was more effective than placebo in alleviating RA symptoms (WMD 4.51, 95% CI [3.00, 6.80], P < 0.00001) and reducing disease activity (WMD 3.10, 95% CI [1.93, 4.97], P < 0.00001). Among the subgroups compared by different doses, only fostamatinib 100 mg bid effectively alleviated disease progression (WMD 4.80, 95% CI [3.14, 7.36], P < 0.00001 for DAS28-CRP < 2.6; WMD 3.41, 95% CI [2.02, 5.77], P < 0.00001 for DAS28-CRP ≤ 3.2) (**Figures 7A** and **8A**).

For the comparison of flexible doses and fostamatinib 100 mg bid, the efficacy of fostamatinib 100 mg bid remained better than the administration of 100 mg bid for 4 weeks followed by 150 mg qd (WMD 0.49, 95% CI [0.29, 0.85], P < 0.00001; **Figure 7B**). Subgroup analyses did not lessen the high level of heterogeneity with different treatment periods. One clinical trial (NCT01197534) was excluded as its effect size was larger than that of other trials, significantly reducing the heterogeneity of DAS28-CRP < 2.6. Nevertheless, for DAS28-CRP ≤ 3.2, there was no difference between fostamatinib 100 mg bid and fostamatinib administered at 100 mg bid for 4 weeks followed by 150 mg qd (WMD 1.11, 95% CI [0.62, 1.99], P = 0.72; **Figure 8B**).

#### DAS28-CRP EULAR Response

**Figure 9:**
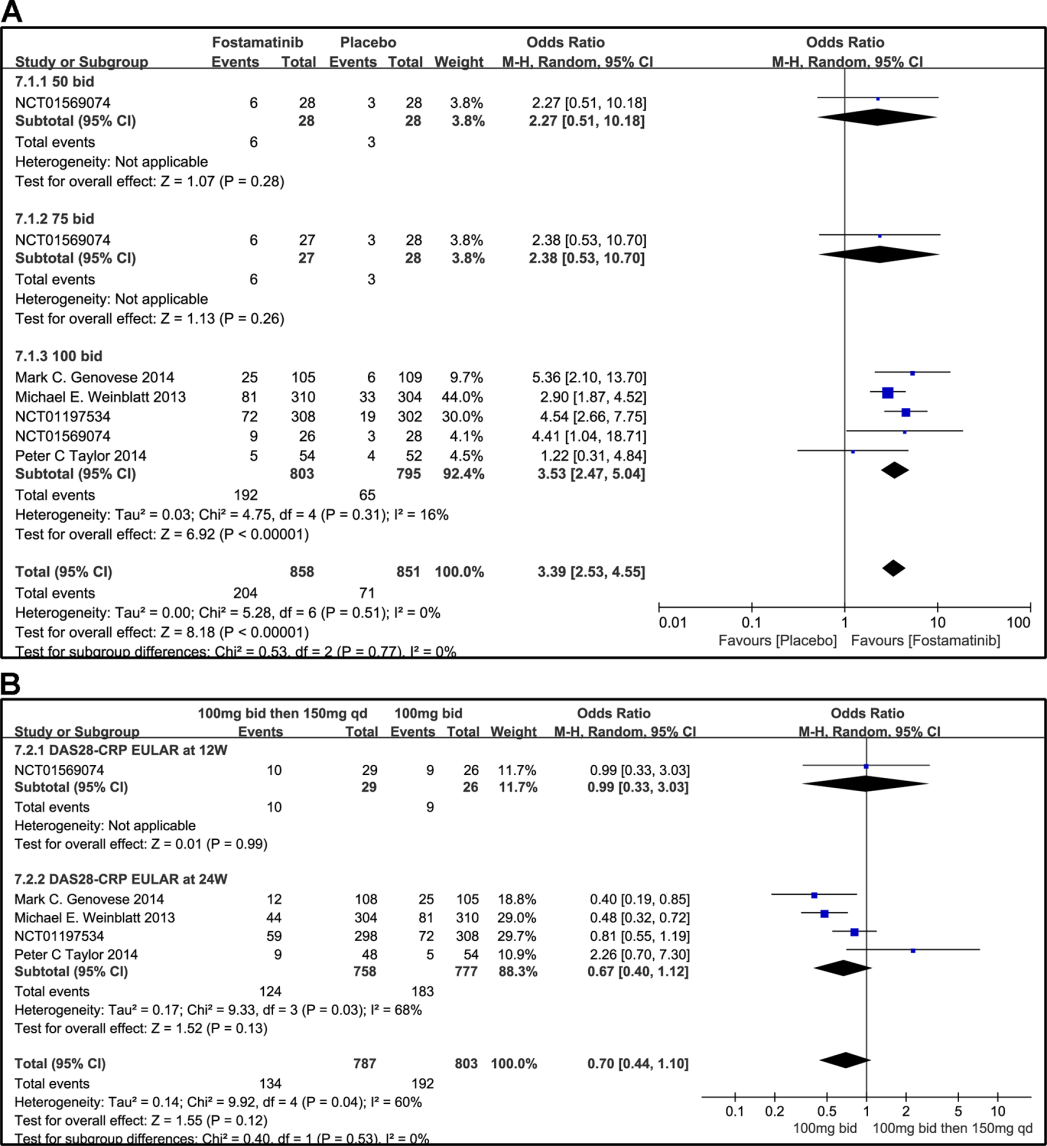
Forest plots for the effect of multiple doses on DAS28-CRP EULAR (only considering good response) at different time points. **(A)** Subgroups administered multiple doses (50, 75, and 100 mg bid) of fostamatinib compared to placebo at 24 weeks from baseline; **(B)** 100 mg bid for 4 weeks followed by 150 mg bid vs. 100 mg bid at 12 and 24 weeks.

According to the EULAR response criteria, the response of fostamatinib was better than that of placebo (WMD 3.39, 95% CI [2.53, 4.55], P < 0.00001; **Figure 9A**), especially fostamatinib 100 mg bid (WMD 3.53, 95% CI [2.47, 5.04], P < 0.00001). However, there was no observable difference between the group administered with fostamatinib 100 mg bid and that administered with fostamatinib 100 mg followed by 150 mg qd (WMD 0.70, 95% CI [0.44, 1.10], P = 0.12; I^2^ = 60%) (**Figure 9B**). Because of the duration of administration, a subgroup analysis was performed; however, a high heterogeneity was still observed at 24 weeks (WMD 0.67, 95% CI [0.40, 1.12], P = 0.13; I^2^ = 68%; **Figure 9B**).

### SF-36

#### SF-36 PCS

**Figure 10:**
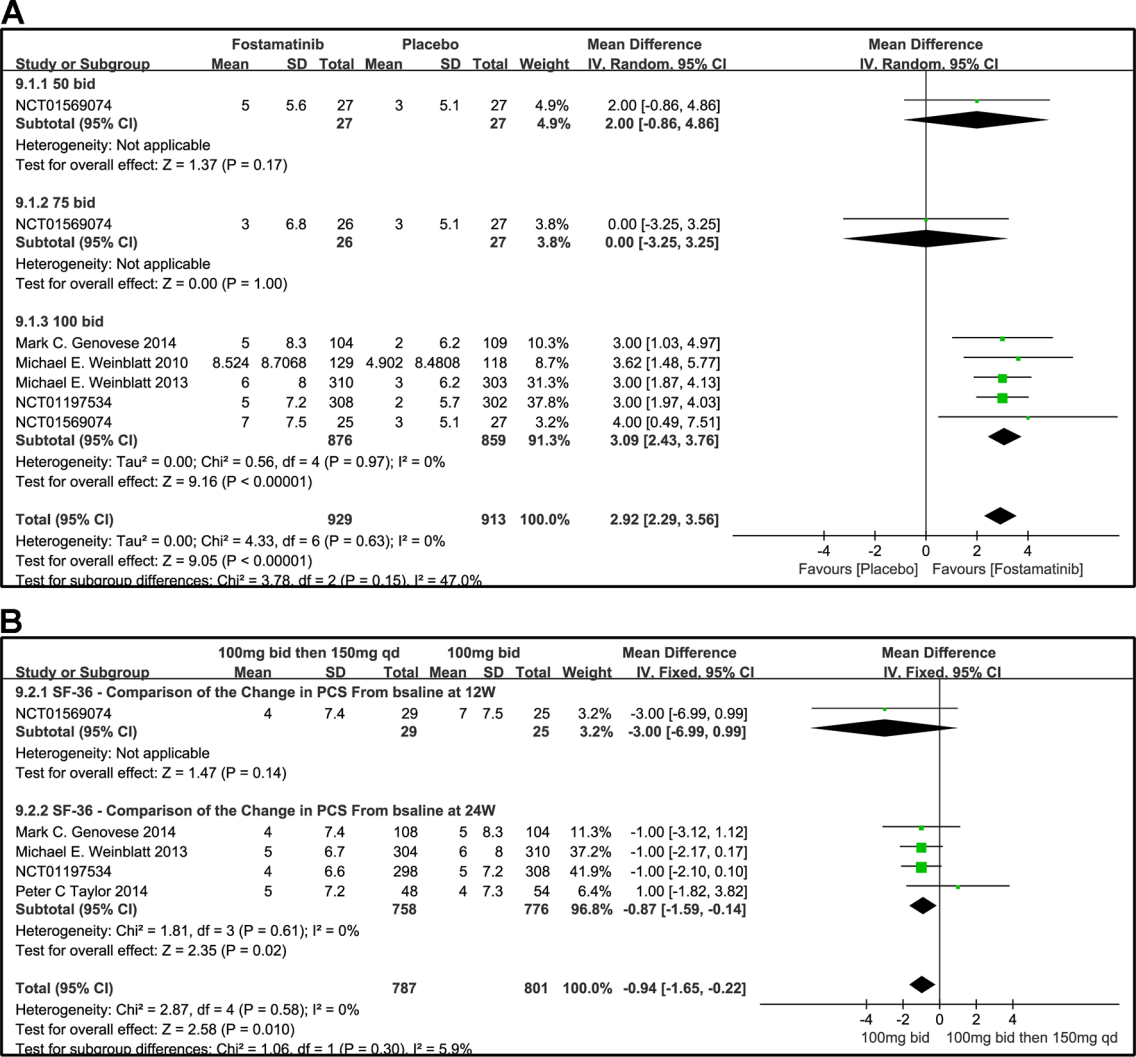
Forest plots for the effect of multiple doses on SF-36 to derive the change in PCS at different time points. **(A)** Subgroups administered multiple doses (50, 75, and 100 mg bid) of fostamatinib vs. placebo at 24 weeks; **(B)** 100 mg bid for 4 weeks followed by 150 mg bid vs. 100 mg bid at 12 and 24 weeks.

The effects of 50, 100, and 75 mg fostamatinib administered bid vs. placebo on the SF-36 PCS change from baseline are shown in **Figure 10A** (WMD 2.92, 95% CI [2.29, 3.56], P < 0.00001, I² = 0%). Administering 100 mg bid (WMD 3.09, 95% CI [2.43, 3.76], P < 0.00001) significantly ameliorated SF-36 PCS but 50 or 75 mg bid could not demonstrate effectiveness (WMD 2.0, 95% CI [-0.86, 4.86], P = 0.17; WMD 0.00, 95% CI [-3.25, 3.25], P = 1). Because of the inconsistency in the effectiveness of different doses, heterogeneity was significantly reduced after removing the subgroup, 75 mg bid; the result of this group also did not differ from the placebo group (WMD 3.04, 95% CI [2.39, 3.68]; I^2^ = 0%; detailed data not shown).

We also compared the effectiveness of a 100 mg bid administration of fostamatinib for 4 weeks followed by 150 mg qd and 100 mg bid at 12 and 24 weeks, as shown in **Figure 10B** (WMD -0.94, 95%CI [-1.65, -0.22], P < 0.00001, I² = 0%). At 24 weeks, the effectiveness of the later regimen improved. A consistency was found with the 100 mg bid regimen (WMD -0.87, 95% CI [-1.59, -0.14], P = 0.02).

#### SF-36 MCS

**Figure 11:**
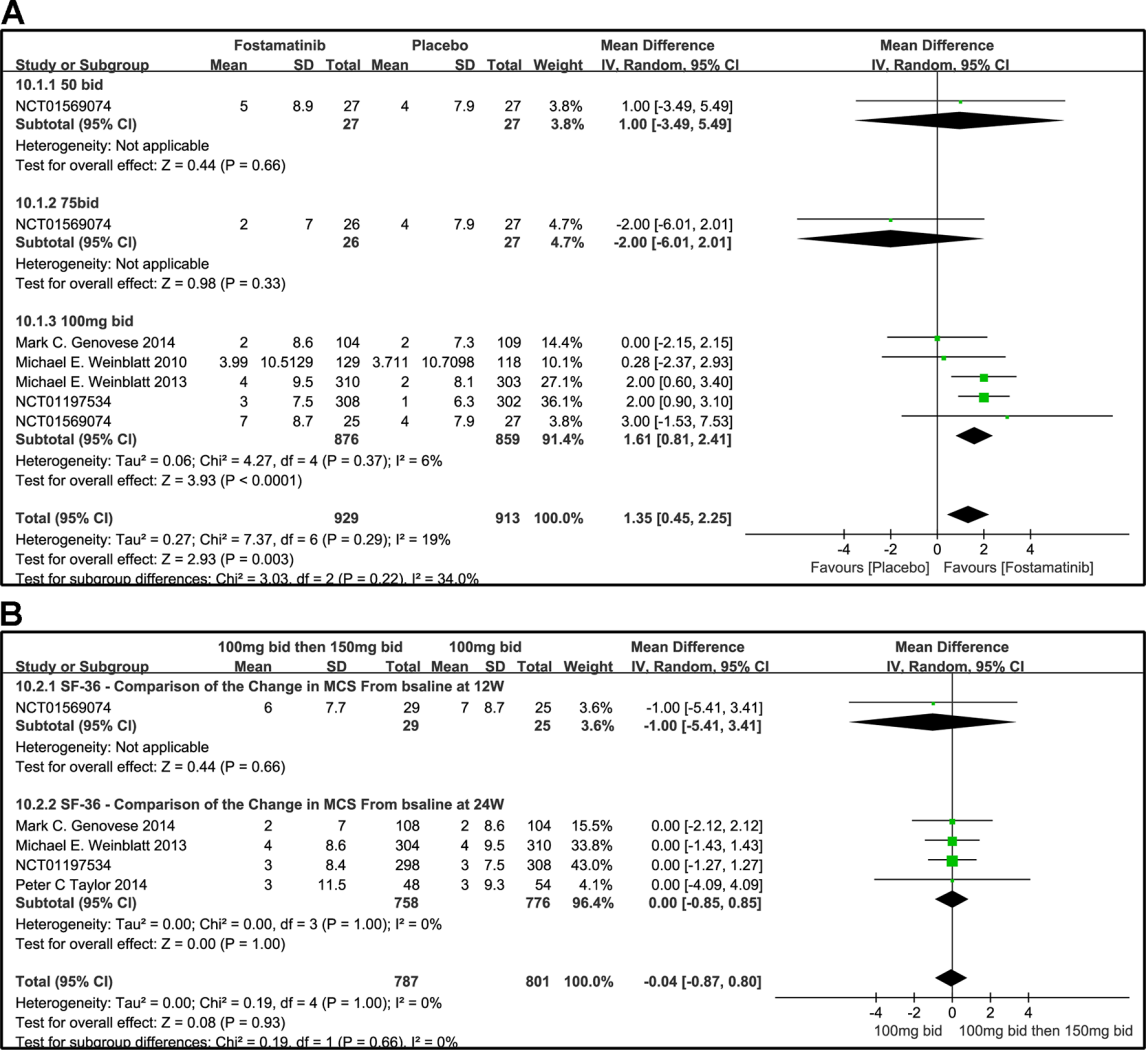
Forest plots for the effect of multiple doses on SF-36 to derive the change in MCS at different time points. **(A)** Subgroups administered multiple doses (50, 75, and 100 mg bid) of fostamatinib vs. placebo at 24 weeks; **(B)** 100 mg bid for 4 weeks followed by 150 mg bid vs. 100 mg bid at 12 and 24 weeks.

The effects of 50, 75, and 100 mg bid fostamatinib vs. placebo on SF-36 MCS change from baseline are shown in **Figure 11A**. The pooled effect estimate of the SF-36 MCS total score was 1.35 (95% CI [0.45, 2.25], P = 0.03, I² = 19%). Intake of 100 mg bid (WMD 1.61, 95% CI [0.81, 2.41], P < 0.0001) resulted in a notably moderate SF-36 MCS. However, 50 mg bid could not demonstrate the effectiveness of fostamatinib (WMD 1.00, 95% CI [-3.49, 5.49], P = 0.66). An opposite outcome was identified with 75 mg bid (WMD -2.00, 95% CI [-6.01, 2.01], P = 0.33). Because of the inconsistency in the effectiveness of different doses, heterogeneity was significantly reduced after removing the 75 mg bid subgroup, which also did not differ from the control group (WMD 1.62, 95% CI [0.88, 0.37]; I^2^ = 0%; detailed data not shown).

**Figure 11B** shows the comparison of the effectiveness of SF-36-MCS between the administration of 100 mg bid for 4 weeks followed by 150 mg qd and 100 mg bid at 12 and 24 weeks. The SF-36-MCS index did not show any difference between the two groups at the time points indicated (WMD -1.00, 95% CI [-5.41, 3.41], P = 0.66; WMD 0.00, 95% CI [-0.85, 0.85], P = 1.00). Likewise, the same results were found for the total score (WMD -0.04, 95%CI [-0.87, 0.80], P = 0.932; I^2^ = 0%).

### HAQ-DI Response

**Figure 12:**
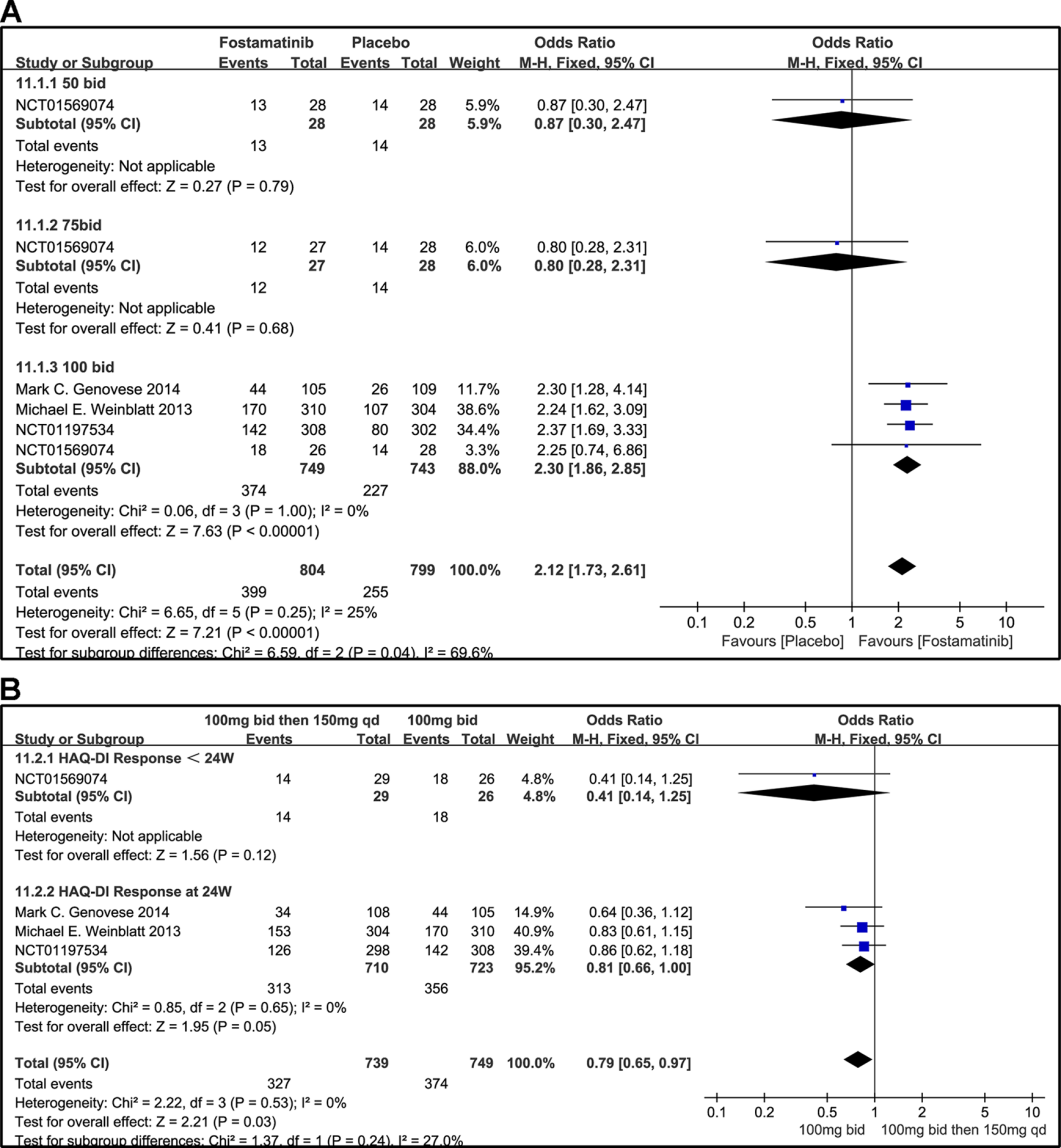
Forest plots for the effect of multiple doses on HAQ-DI response at different time points. **(A)** Subgroups administered multiple doses (50, 75, and 100 mg bid) of fostamatinib vs. placebo at 24 weeks; **(B)** 100 mg bid for 4 weeks followed by 150 mg bid vs. 100 mg bid < 24 and 24 weeks.

When the dosages of 50 and 75 mg bid were administered, a significant difference was not found between the two groups (OR 0.87, 95% CI [0.30, 2.47], P = 0.79; OR 0.80, 95% CI [0.28, 2.31], P = 0.68). For HAQ-DI response, a significant decrease from baseline was observed in the 100 mg bid group compared to placebo (OR 2.30, 95% CI [1.86, 2.85], P < 0.00001). Overall OR was 2.12 (95% CI [1.73, 2.61], P < 0.00001, I² = 25%; **Figure 12A**).

When 100 mg bid of fostamatinib was administered for 4 weeks followed by 150 mg qd or 100 mg bid, a significant difference at < 24 or 24 weeks (OR 0.41, 95% CI [0.14, 1.25], P v= 0.12; OR 0.81, 95% CI [0.66, 1.00], P = 0.05; **Figure 12B**) was not observed.

### Safety and Tolerability

#### SAEs

**Figure 13:**
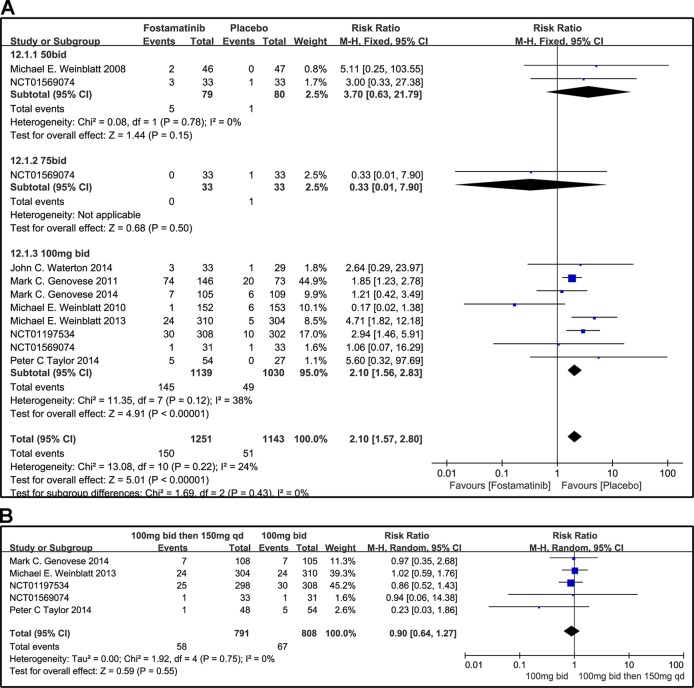
Forest plots for the effect of multiple doses on SAEs. **(A)** Subgroups administered multiple doses (50, 75, and 100 mg bid) of fostamatinib vs. placebo; **(B)** 100 mg bid for 4 weeks followed by 150 mg bid vs. 100 mg bid.

Overall, the incidence of SAEs of fostamatinib was greater than that with placebo (RR 2.10, 95% CI [1.57, 2.80], P < 0.00001; **Figure 13**). In addition, SAEs could be observed with the 50 mg bid regimen (RR 3.70, 95% CI [0.63, 21.79], P = 0.15). Because of the small sample size of the group administered the 75 mg bid dosage regimen, SAEs could not be fully determined in this group (RR 0.33, 95% CI [0.01, 7.90] P = 0.5). For overall AEs, a significant difference was found between fostamatinib 100 mg bid and placebo (RR 2.10, 95% CI [1.56, 2.83], P < 0.00001; **Figure 13A**). Heterogeneity was significantly reduced after the removal of a study (Weinblatt et al., 2010) (detailed data not shown). The risk of SAEs was similar between the administration of 100 mg bid for 4 weeks followed by 150 and 100 mg bid (RR 0.90, 95% CI [0.64, 1.27], P = 0.55; **Figure 13B**).

#### Other AEs

**Figure 14:**
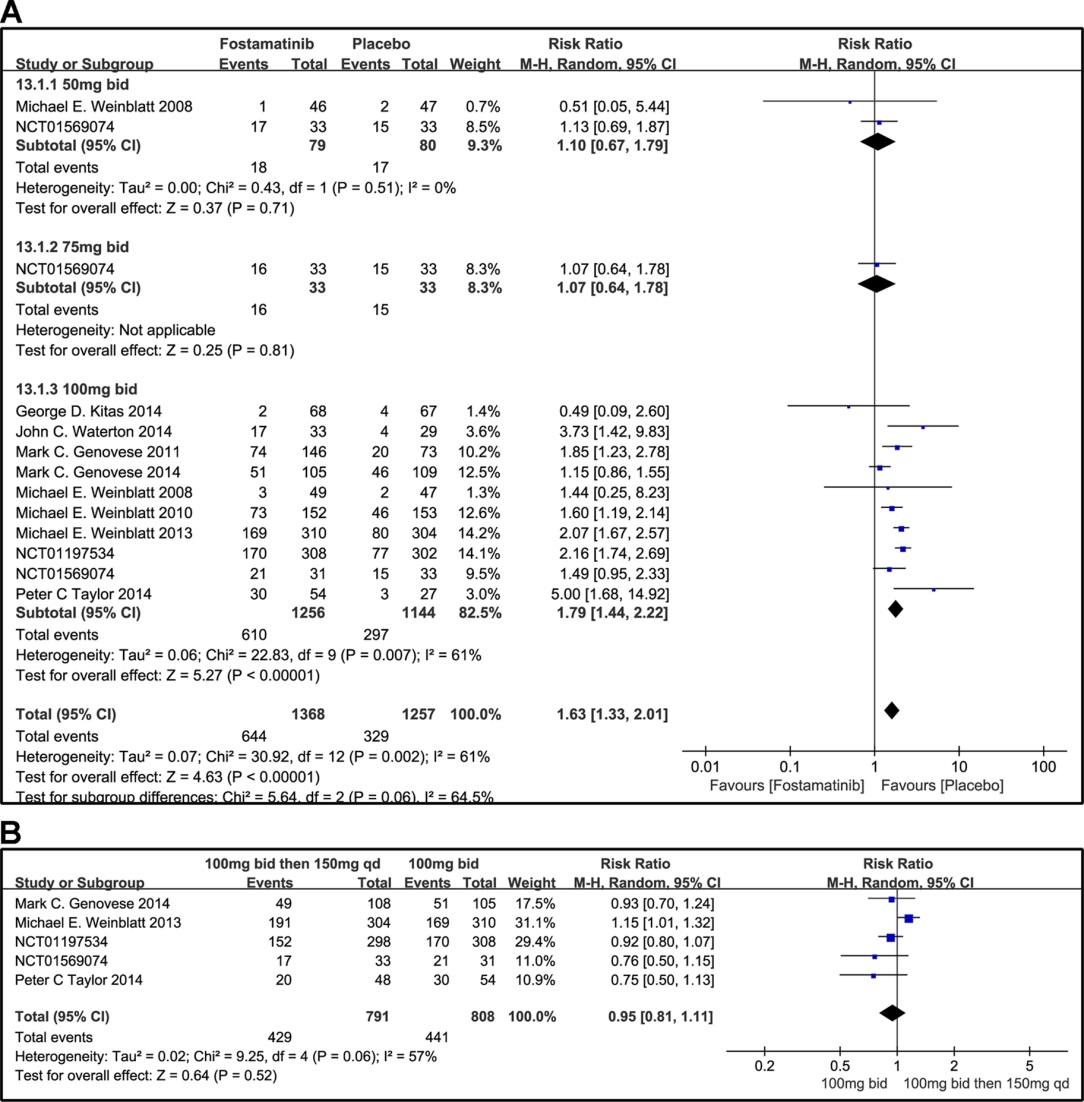
Forest plots for the effect of multiple doses on other AEs. **(A)** Subgroups administered multiple doses (50, 75, and 100 mg bid) of fostamatinib vs. placebo; **(B)** 100 mg bid 4 weeks followed by 150 mg bid vs. 100 mg bid.

When 50 or 75 mg bid was administered, there was no significant difference between fostamatinib and placebo in overall other AEs (RR 1.10, 95% CI [0.67, 1.79], P = 0.71; RR 1.07; 95% CI [0.64, 1.78], P = 0.81; **Figure 14A**). However, when 100 mg bid was administered, the probability of the occurrence of other AEs was higher than that of placebo (RR 1.79, 95% CI [1.44, 2.22], P < 0.00001). Though it is imperative to remove possible effect sizes to avoid heterogeneity, the heterogeneity is still greater than 50%. When 100 mg bid was administered for 4 weeks followed by 150 mg qd and compared to the administration of 100 mg bid, a significant difference was not found between the two dosing regimens (RR 0.95, 95% CI [0.81, 1.11], P = 0.52; **Figure 14B**).

## Discussion

### Summary of Main Findings

RA is a chronic inflammatory joint disease that can result in damages to the cartilage and bone as well as disability (Smolen et al., 2016). The Syk inhibitor, fostamatinib, has been reported to be effective for the treatment of RA. Therefore, in this systematic review and meta-analysis, we discussed the safety and efficacy of multiple dosages of fostamatinib. This meta-analysis was performed according to the methods in the Cochrane handbook and the PRISMA Statement protocol. The applied search strategy revealed all relevant published and unpublished articles but we excluded an open label study with five healthy subjects and two pharmacokinetics studies from our analysis.

Among the results acquired from the 11 RCTs in the present meta-analysis, we believe that ACR20 and DAS28-CRP < 2.6 are a primary goal in active RA control. They both had a positive change at 12 weeks. The therapeutic effect of 100 mg bid 4 weeks then 150 mg qd is not as good as that of 100 mg bid at 24 weeks. In addition, we observed high heterogeneity when the 100 mg bid dose was compared in the ACR20 group, especially in the 100 mg bid subgroup. After removing the study by Genovese et al. (2011), the heterogeneity was observed to decrease significantly. After the removal of “NCT01197534”, such trend was also reflected in DAS28-CRP < 2.6 as high total heterogeneity was found in DAS28-CRP < 2.6 at 12 weeks. We speculate that the high level of heterogeneity is caused by the better efficacy of the study compared to the other studies; thus, when removed, heterogeneity can be reduced. This creates doubt in the credibility of the data.

The results acquired from the 11 RCTs in the present meta-analysis indicate that fostamatinib yielded a better ACRn score than placebo. Nonetheless, the same phenomenon can be seen for the indexes ACR50, ACR70, DAS28-CRP ≤ 3.2, and DAS28-CRP EULAR response. We can also confirm that 100 mg bid is the optimal choice for RA patients. Furthermore, SF-36-PCS, MCS and HAQ-DI response had positive change. At 24 weeks, 100 mg bid was more effective than 100 mg bid followed by 150 qd in SF-36-PCS. However, remission was not observed in SF-36-MCS. This is a reminder that physical and mental components are also contributors. Taken together, fostamatinib is a reliable, effective, and potential drug for the clinical treatment of RA. The results of this meta-analysis also indicate that fostamatinib 100 mg bid is the optimal dose recommended for clinical use, aligning with the results of a previous study (Kunwar et al., 2016).

Our current systematic review and meta-analysis included phase II and III studies where treatment outcome with fostamatinib was compared to that of placebo. Adverse reactions caused by 50 and 75 mg bid resulted in no significant difference at 12 weeks; this is for SAEs or other AEs. However, there were significant differences in adverse reactions between 100 mg bid group and the placebo group. This might be because the former has a relatively small sample size and low degree of credibility, or because of its inability to verify the cumulative adverse reactions in the later period of medication without additional studies assessing the long-term adverse reactions of the medication for 24 weeks or more.

Based on the RCTs, incidence of SAEs was mainly due to gastrointestinal disorders, hepatobiliary disorders, infections and infestations, and musculoskeletal and connective tissue disorders. However, a marked increase in the frequency of serious infections and infestations were not found in the fostamatinib group compared to the placebo group. Other AEs mainly included hypertension, diarrhea, nausea, ALT increase, nasopharyngitis, vomiting, dyspepsia, headache, dizziness, arthralgia, and flatulence, etc. Kitas et al. (2014) revealed that fostamatinib causes small but significant elevations in the 24 h mean ambulatory systolic blood pressure and diastolic blood pressure of patients with active RA. The overall safety profile of fostamatinib, when administered at either 100 mg bid for 4 weeks followed by 150 mg qd or 100 mg bid, was generally consistent with that observed in patients treated *via* both schedules. Taylor et al. (2015) suggested that fostamatinib monotherapy demonstrated inferior response to adalimumab at 24 weeks. This highlights the need for more research to confirm the efficacy of fostamatinib compared to other protease inhibitors and/or combination of other drugs. A long-term follow-up study is also necessary to determine the effectiveness of fostamatinib.

### Limitations

This meta-analysis had some limitations. First, six of the 11 clinical trials did not clearly describe the allocation concealment, which demonstrated low or very low confidence in GRADE estimates for all outcomes (**Supplementary Table 2**). Therefore, we implore researchers and authors to perform an in-depth recording of their experimental methods in future clinical trials and publications to enable readers and reviewers to better understand the specific contents of the experiments. Second, we observed significant statistical heterogeneity in some of the outcomes. Thus, we explored the sources of heterogeneity by subgroup analysis and removed inconsistencies in GRADE assessments for unexplained heterogeneity. Third, some results were not entirely accurate because of the small study population. Consequently, additional trials that are well-designed and conducted are urgently required to confirm our findings.

### Conclusion

In summary, the results of the present systematic review and meta-analysis demonstrate that 100 mg bid of fostamatinib displays a greater efficacy than placebo. This is evident by the clinically meaningful reduction in the scores of patients with active RA who exhibit either an inadequate response or a lack of response to MTX, DMARD, or TNF-α antagonist. An economic evaluation of the pharmacodynamics of fostamatinib is still required, as well as an effective method to illustrate the problem and results in a comprehensive and adequate manner. These achievements would ultimately provide a more economical and reasonable scheme for the treatment of patients with RA.

## Data Availability

All datasets generated for this study are included in the manuscipt and/or **Supplementary files**.

## Author Contributions

YK, XJ, YY, and JW conceived and designed the study. YK, XJ, DQ, LW, JY, AW, FH, YY, and JW reviewed the literature. YK, XJ, YY, and JW wrote the manuscript.

## Funding

This work was supported by grants from the National Natural Science Foundation of China (Nos. 81774013 and 81804221), the Science and Technology Planning Project of Sichuan Province, China (Grant Nos. 2018JY0237 and 2019YJ0484), Educational Commission of Sichuan Province, China (Grant No. 18TD0051), Administration of traditional Chinese medicine in Sichuan Province, China (Grant Nos. 2018JC013 and 2018JC038), and the Science and Technology Program of Luzhou, China [Grant Nos. 2017-S-39(3/5) and 2016LZXNYD-T03].

## Conflict of Interest Statement

The authors declare that the research was conducted in the absence of any commercial or financial relationships that could be construed as a potential conflict of interest.
