# Supplementary material for: Efficacy and Safety of Multiple Dosages of Fostamatinib in Adult Patients With Rheumatoid Arthritis: A Systematic Review and Meta-Analysis
Source: Front Pharmacol. 2019 Aug 14;10:897. doi: 10.3389/fphar.2019.00897 (PMC6702340; doi:10.3389/fphar.2019.00897)
Supplement: Supplementary file 1 [file DataSheet_1.docx]

Supplementary Material

# Supplementary Table 1

**Table 1** Patient Characteristics of Included Studies.

| **Study&year** | **Age,**  **years(M±SD)** | **Gender** | **Race** | **Background therapy** | **Location** | **Sponsor** |
| --- | --- | --- | --- | --- | --- | --- |
| Michael E. Weinblatt *et al* (2014) | 52 ± 12.0 | F: 83.9%  M: 16.1% | White：69.7%; American：2.7%; Asian：2.0%; Native American or Alaska Native：4.0%; Indian or Pakistani：5.8%; Other：15.8% | Receiving MTX but not responding | United States, Australia, Europe, South America, Asia | AstraZeneca |
| *NCT 01197534* | 53 ± 11.9 | F: 81.7%  M: 18.3% | White：80.4%; Black or African American: 4.0%; Asian: 6.7%;Native American or Alaska Native：0.4%; Indian or Pakistani：11.5%; Other：17.8% | Receiving DMARD but not responding | United States, Canada, Europe, South Africa, Asia | AstraZeneca |
| Mark C. Genovese  *et al* (2014) | 53 ± 12.3 | F: 81.1%  M: 18.9% | White: 83.5%; Black or African American：7.1%; Asian：0.3%; Native American or Alaska Native：1.9%; Indian or Pakistani：0.9%; Other：6.2% | Receiving MTX and inadequate response to a single TNF-alpha antagonist | United States, Canada, Europe, South America, South Africa | AstraZeneca |
| Peter C Taylor  *et al* (2014) | 50 ± 11.8 | F: 79.2%  M: 20.8% | White：90.6%; Black or African American：6.0%; Asian：1.5%; Native American or Alaska Native：0.4%; Indian or Pakistani：0.8%; Other：0.8% | DMARD intolerant or inadequate response | United States, Canada, Europe, South Africa | AstraZeneca |
| John C. Waterton  *et al* (2017) | 51 ± 12.9 | F: 72.2%  M: 27.8% | White：75.6%; Black or African American：7.8%; Indian or Pakistani：10.0% | DMARD intolerant or inadequate response | United States, Canada, Europe, South Africa | AstraZeneca |
| *NCT 01569074* | 53 ± 11.9 | F: 87.1%  M: 12.9% | Asia: 100% | Receiving MTX but not responding | Asia | AstraZeneca |
| George D. Kitas  *et al* (2014) | 54 ± 12.5 | F: 84.4%  M: 15.6% | White：89.6%; Black or African American：8.1%; South Asia：2.2% | Receiving DMARD | United States, Europe, South Africa | AstraZeneca |
| Michael E. Weinblatt  *et al* (2008) | 52.3 ± 10.6 | F: 86.8%  M: 13.2% | White：40.2%; Hispanic：54.5% | Receiving MTX | United States, Mexico | Rigel Pharmaceuticals |
| Michael E. Weinblatt  *et al* (2010) | 52.5 ± 12.8 | F: 85.3%  M: 14.7% | White：44.6%; Hispanic：51.9% | Receiving MTX but not responding | United States, Mexico, Europe | Rigel Pharmaceuticals |
| Michael E. Weinblatt  *et al* (2013) | 52.5 ± 12.8 | F: 85.3%  M: 14.7% | White：44.6%; Hispanic：51.9% | Receiving MTX but not responding | United States, Mexico, Europe | Rigel Pharmaceuticals |
| Mark C. Genovese  *et al* (2011) | 56.0 ±11.7 | F: 80.4%  M: 19.6% | Hispanic:30.6% | 'Failed' a biologic therapy | United States, Europe | Rigel Pharmaceuticals |

Note: NA: Not available; MTX: methotrexate; DMARD: disease modifying anti-rheumatic drug

# Supplementary Table 2

**Table 2.** Quality of included studies, assessed using the GRADE system.

| **Quality assessment** | | | | | | | **No of patients** | | **Relative (95% CI)** | **Quality** | **Importance** |
| --- | --- | --- | --- | --- | --- | --- | --- | --- | --- | --- | --- |
| **No of studies** | **Design** | **Risk of bias** | **Inconsistency** | **Indirectness** | **Imprecision** | **Other considerations** | **ACR20-new** | **Control** |  |  |  |
| **ACR20**  8 | randomised trials | Serious^1^ | Serious^2^ | Serious^3^ | no serious imprecision | None^4^ | 576/1250  (46.1%) | 354/1171  (30.2%) | OR 1.96 (1.46 to 2.61) | ⊕OOO VERY LOW | CRITICAL |
| **ACR50**  8 | randomised trials | Serious^1^ | no serious inconsistency^2^ | Serious^3^ | no serious imprecision | None^4^ | 313/1250  (25%) | 157/1169  (13.4%) | OR 1.67 (0.97 to 2.88) | ⊕⊕OO LOW | CRITICAL |
| **ACR70**  8 | randomised trials | Serious^1^ | no serious inconsistency | Serious^3^ | no serious imprecision | None^4^ | 164/1250  (13.1%) | 81/1169  (6.9%) | OR 1.71 (0.74 to 3.97) | ⊕⊕OO LOW | CRITICAL |
| **ACRn**  7 | randomised trials | Serious^1^ | Serious^2^ | Serious^3^ | no serious imprecision | None^4^ | 1112 | 1031 | - | ⊕OOO VERY LOW | CRITICAL |
| **DAS28-CRP<2.6**  6 | randomised trials | Serious^1^ | Serious^2^ | Serious^3^ | no serious imprecision | None^4^ | 136/990  (13.7%) | 39/935  (4.2%) | OR 3.23 (1.6 to 6.53) | ⊕OOO VERY LOW | CRITICAL |
| **DAS28-CRP<3.2**  4 | randomised trials | Serious^1^ | no serious inconsistency | Serious^3^ | no serious imprecision | None^4^ | 81/358  (22.6%) | 39/313  (12.5%) | OR 1.85 (0.84 to 4.08) | ⊕⊕OO LOW | CRITICAL |
| **DAS28-CRP EULAR**  4 | randomised trials | Serious^1^ | no serious inconsistency | Serious^3^ | no serious imprecision | None^4^ | 199/804  (24.8%) | 67/799  (8.4%) | OR 3.56 (2.64 to 4.8) | ⊕⊕OO LOW | IMPORTANT |
| **SF-36 PCS**  5 | randomised trials | Serious^1^ | no serious inconsistency | Serious^3^ | no serious imprecision | None^4^ | 929 | 913 | - | ⊕⊕OO LOW | IMPORTANT |
| **SF-36 MCS**  5 | randomised trials | Serious^1^ | no serious inconsistency | Serious^3^ | no serious imprecision | None^4^ | 929 | 913 | - | ⊕⊕OO LOW | IMPORTANT |
| **HAQ-DI respons**e  4 | randomised trials | Serious^1^ | no serious inconsistency | Serious^3^ | no serious imprecision | None^4^ | 399/826  (48.3%) | 255/799  (31.9%) | OR 2.02 (1.65 to 2.47) | ⊕⊕OO LOW | IMPORTANT |
| **SAEs**  9 | randomised trials | Serious^1^ | no serious inconsistency | Serious^3^ | no serious imprecision | None^4^ | 150/1251  (12%) | 47/1067  (4.4%) | RR 2.22 (1.65 to 2.97) | ⊕⊕OO LOW | CRITICAL |
| **Other Aes**  9 | randomised trials | Serious^1^ | Serious^2^ | Serious^3^ | no serious imprecision | None^4^ | 628/1279  (49.1%) | 299/1086  (27.5%) | RR 1.64 (1.31 to 2.05) | ⊕OOO VERY LOW | CRITICAL |

Note: ^1^ Lack allocation concealment
^2^ Relatively high heterogenicity
^3^ Indirect comparison
^4^ Corporate sponsors
